# Annotations

**Published:** 1905-04-15

**Authors:** 


### ANNOTATIONS.

### The Sale of Hypodermic Syringes.

THE habit of self-medication is a practice which has no defenders. Even those who indulge themselves in this direction prefer for the most part to conceal their peculiarity and will apologise in a somewhat shamefaced manner should the secret leak out. More especially does a general condemnation apply to the self-use of powerful narcotic drugs; and whilst medical practitioners have special experience of this evil there are unfortunately only too many members of the public who have learned of its disasters from the habits of relatives and friends. Through the progress of the pharmaceutical art many preparations now exist which offer a ready temptation to those disposed to seek the fallacious solace of narcotics, and some of these can be easily procured. Compressed doses of powerful drugs are often prominently displayed in chemists' shops, and there is little or no restraint on their purchase. It is well known that self-medication by the hypodermic method is one of the most insidious and intractable forms of the drug habit, and any proposal which would render this practice difficult or impossible would be in the public interest. Hence we gladly give credit to a leading Glasgow pharmacist who at a recent meeting of chemists and druggists protested against the sale to the public either of hypodermic syringes or of tablets for the preparation of hypodermic injections. The advice that chemists should refuse to supply these articles except in response to an order from a medical practitioner indicates an appreciation of responsibility and public duty that is worthy of general commendation. Certainly, apart from medical advice, the hypodermic syringe has no honest purpose, and the State might well forbid its purchase unless sanctioned by medical authority. In the meantime we will commend to pharmacists the advice and example of their Glasgow confrère.

### London's Lunatics.

IT is curious to notice how little interest is taken by the general public in anything connected with the care of the insane. Not even the great increase in the numbers of the registered insane, nor their ever-increasing cost to the community can obtain more than a passing glance. But the importance of the subject is well illustrated in an article by Dr. Jones, the medical superintendent of the Claybury Asylum, in the current number of the *Westminster Review*. Dr. Jones points out that on May 1st, 1890, when the new London County Council took over the care of the insane from the county justices there were only four asylums containing an aggregate of 7,240 patients, whereas there are now nine asylums containing an aggregate of 16,539 patients; and in addition to that number there are 900 lunatics boarded out in asylums not under control of the Council. We thus get 17,439 patients; but even that is not the total of London's lunatics, for to it must be added a few thousand more to include those confined in the Metropolitan Asylums at Leavesden, Caterham, and Darenth, and there are a few hundreds chargeable to the City of London in the asylum at Dartford. A new asylum for 2,000 patients is being built near Epsom, and, as the pauper lunatics of the administrative County of London increase by 500 a year, an asylum of like size will have to be built every four years to accommodate this ever-growing army of pauper insane. It is quite clear that if this rapid increase is to be arrested it must be by prevention and not by cure. There is no doubt that hereditary tendency and alcohol account together for 50 per cent. of the insanity we see around us; and it is surely time that some serious attempt was made to limit these causes, even although the "sacred liberty" of the subject be interfered with to some extent. We should like to see Dr. Jones's article reprinted and distributed in pamphlet form; and if the author will act upon this suggestion we hope he will add an exhaustive paragraph on aliens in our county asylums. It is well known that some of the London asylums contain large numbers of these undesirable and heterogeneous elements.

### Lessons from Without.

IN a recent lecture addressed to the students of an important metropolitan school of medicine a well-known physician, after speaking of the success of many of the medicinal preparations so largely advertised in the lay press, had the courage to suggest to his hearers that the profession might possibly learn something from the method and fashion of the so-called secret remedy. He pointed out that many of these articles are directed to relieve the minor ailments of mankind, which are none the less real because they cannot be included within the scheme of a scientific pathology. On the other hand, the medical practitioner is perhaps apt at times to pay too small an amount of attention to these slighter disorders and to be attracted by conditions which are capable of exact and certain diagnosis. The adjustment and combination of medicines for the relief of troublesome symptoms and the presentation of remedies in a not unpleasant form are ends well worthy of careful study. They mean the diminution of suffering, and, though this is of minor importance, they are not inconsiderable factors in the attainment of success in practice. If help towards these ends can be obtained it is welcome, come from what quarter it may. Naturally the medical profession, conscious of the time and effort needed to acquire a real knowledge of disease, looks with a somewhat intolerant eye on the haphazard and blind suggestions of those who have no pretence to possess this knowledge. Still it must be recognised that amidst a thousand foolish proposals, one here and there has had a basis of reason and common-sense. Until a year or two ago the military art and the promotion of individual muscular efficiency were deemed to have a secure position in the traditions and practices of the West. Yet recent events in the Far East have shown that knowledge and skill in both of these directions can be increased from very unexpected sources. For those who dwell in an orthodox tabernacle there yet may be lessons to be learned from the voices that are outside.

